# OTUB1 suppresses Hippo signaling via modulating YAP protein in gastric cancer

**DOI:** 10.1038/s41388-022-02507-3

**Published:** 2022-10-21

**Authors:** Cheng Yan, Huijie Yang, Peng Su, Xin Li, Zhongbo Li, Dehai Wang, Yifeng Zang, Tianshi Wang, Ziping Liu, Zhuocong Bao, Shuxiao Dong, Ting Zhuang, Jian Zhu, Yinlu Ding

**Affiliations:** 1 School of Medicine, Key Laboratory of Nano-carbon Modified Film Technology of Henan Province, Diagnostic Laboratory of Animal Diseases, Xinxiang University, Xinxiang, 453003 Henan China; 2 Xinxiang Key Laboratory of Tumor Migration and Invasion Precision Medicine, School of Laboratory Medicine, Xinxiang Medical University, Xinxiang, 453003 Henan Province China; 3 Department of Pathology, Qilu Hospital, Cheeloo College of Medicine, Shandong University, Jinan, 250033 China; 4 Department of General Surgery, The Second Hospital, Cheeloo College of Medicine, Shandong University, Jinan, Shandong Province China; 5 Department of Gastroenterology Surgery, Shandong Provincial Third Hospital, Jinan, 250000 China; 6 Central Hospital Affiliated to Shandong First Medical University, Shandong First Medical University & Shandong Academy of Medical Sciences, Jinan, 250013 China

**Keywords:** Oncogenes, Gastric cancer, Growth factor signalling

## Abstract

Gastric cancer is one of the most lethal human malignancies in the world. Although great efforts are put in developing novel therapeutic targets, the effective targeting drugs are still limited. Recent studies reveal the abnormality of Hippo/YAP axis play critical role in the oncogenic process of gastric cancer. It is of great importance to demonstrate the regulation of Hippo signaling activity and YAP protein turnover in gastric cancer. Besides, the phosphorylation cascade on YAP function, which has been thoroughly investigated, the ubiquitination of YAP is also important in Hippo signaling status. Here, We utilized the DUB (Deubiquitinase) siRNA library to identify critical DUB for Hippo signaling. We discovered OTUB1 as a critical factor to facilitate gastric cancer cell stemness and progression, which deubiquitinated and stabilized YAP protein. The clinical data analysis implicated OTUB1 was higher expressed in gastric cancer, which correlated with YAP activity and poor survival. OUTB1 interacted with YAP protein via its OTU domain (Ovarian tumor domain) and deubiquitinated YAP at several lysine sites (K90, K280, K343, K494 and K497), which subsequently inhibited YAP degradation. Our study revealed a novel deubiquitinase of Hippo/YAP axis and one possible therapeutic target for YAP-driven gastric cancer.

## Introduction

The Hippo signaling pathway plays essential roles in several biological processes, including organ size control, tissue homeostasis, carcinogenesis and immune response [1, 2]. The activation of Hippo signaling is subject to a phosphorylation cascade, in which the mammalian Hippo kinases MST1/2 phosphorylates the LATS1/2 kinase, while the phosphorylated LATS1/2 promotes the phosphorylation of YAP/TAZ protein, leading the localization in the cytosol and protein degradation [3]. If the Hippo signaling is inactivated, the YAP/TAZ are unphosphorylated and translate into the nuclear to promote their target genes expression [4]. In human cancers, YAP protein was elevated in several human cancers, including gastric cancer, while the expression level of YAP is correlated with distant metastasis and poor survival [5]. The molecular studies revealed that YAP could promote gastric cancer invasion and migration [6]. Besides, the activation of Hippo/YAP axis could synergize with interferon-IRF3 pathway to promote cancer progression in EBV infected gastric cancer models [7]. Based on these backgrounds, we can conclude the hyper-activation of Hippo-YAP axis plays important roles in carcinogenesis of gastric cancer, while targeting YAP protein function or expression could be a promising strategy for gastric cancer.

Although, YAP protein level and activity is tightly controlled by a series of Hippo signaling kinases in physiological conditions, it is mysterious that YAP protein is overexpressed or hyper-activated in malignancies, even the phosphorylation feedback loop by MST1/2 and LATS1/2 are still functional [8, 9]. The phosphorylation cascade of MST1/2-LATS1/2-YAP has been proved to play central roles in regulation Hippo/YAP activity, but recent studies reveal several other post-translational modifications, such as ubiquitination, also exert the important functions on Hippo signaling [10, 11]. The ubiquitination modification is a reversible process, which is subject to a delicate balance between E3 ubiquitin ligases and deubiquitinases [12, 13]. The DUBs mainly cleave the ubiquitin from the substrates and counteract with ubiquitin ligases for protein degradation. There are about 100 DUBs existed in human genome, while the USP (Ubiquitin Specific Protease) family is the biggest [14]. Although several DUB family members were reported to modulate Hippo/YAP activity and human cancer progression, it is still not totally clear which of the DUBs are the critical modulators in Hippo signaling and cancer progression. In the current study, we try identify the most important DUBs for Hippo/YAP activity and gastric cancer progression, which makes great clinical impacts and significances for gastric cancer therapeutics.

*OTUB1* (OUT Deubiquitinase Ubiquitin Aldehyde Binding 1) belongs to the ovarian cancer protease family member, which is comprised of 271 amino acids [15]. Previous studies demonstrated that OTUB1 participated in several physiological processes, including immune response and ferroptosis [16, 17]. In cancers, OTUB1 was proved to promote cancer progression via stabilizing ATF6 protein and PDL1 for cancer cell immune evasion [18]. In our study, our siRNA screening data indicated the critical role of OTUB1 in Hippo signaling in gastric cancer. OTUB1 supported gastric cancer proliferation and metastasis via enhancing Hippo/YAP axis activity. Our study proposed a non-genomic regulation between OTUB1 and Hippo/YAP axis and a promising target for gastric cancer treatments.

## Result

### OTUB1 is an important regulator in Hippo signaling function in gastric cancer

**Fig. 1:**
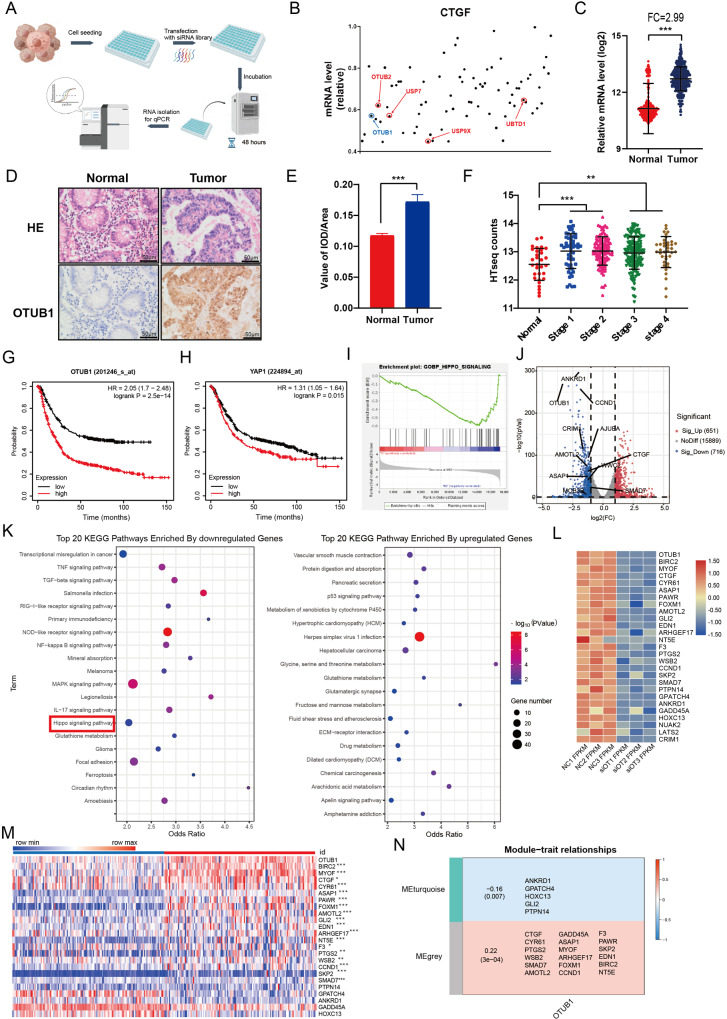
Identification of OTUB1 as a key regulator of Hippo signaling pathway in gastric cancer. **A** Flowchart of screening critical DUBs of modulating Hippo signaling in HEK293. **B** The relative expression level of CTGF in HEK293 cells transfected with DUBs in the screening library. The RT-qPCR results is normalized to 1. Blue dot represents OTUB1 and red dot represents previous reported DUBs regulating Hippo pathway. **C** The relative RNA level of OTUB1 in gastric tumor samples versus normal samples in TCGA combined with GTEX are shown. **D** Immunohistochemistry (IHC) detecting OTUB1 expression in gastric cancer specimen as well as matched normal tissues. **E** Quantitative IOD values for IHC staining between normal and tumor tissues are presented in the histogram. Two-tailed paired Student’s *t*-test is conducted to detect significant differences between normal and tumor tissues ^***^P < 0.001). **F** Quantitative HTseq counts of OTUB1 between normal tissues and different stage of tumor are presented in the histogram. One-way ANOVA is conducted to detect significant differences (^**^P < 0.01, ^***^P < 0.001). **G** Kaplan–Meier survival analysis comparing the overall survival of gastric cancer patients with high and low OTUB1 expression in KMPLOT database. **H** Kaplan–Meier survival analysis comparing the overall survival of gastric cancer patients with high and low YAP expression in KMPLOT database. **I** Gene Set Enrichment Analysis (GSEA) of RNA-Seq data from AGS cell lines treated with scrambled or two independent siRNA targeting OTUB1 (*P* < 0.001). **J** Volcano map of RNA-seq data from AGS cell lines treated with scrambled or two independent siRNA targeting OTUB1. | log2Fold change | >1 and adj. *P* value < 0.05 are set as screening criteria. **K** Top 20 KEGG pathway enriched by differentially down-regulated genes (left panel) or up-regulated genes (right panel). **L** Heatmap of differentially Hippo pathway related genes in RNA-seq data from AGS celllines treated with scrambled or two independent siRNA targeting OTUB1. **M** Heatmap of OTUB1 and differentially Hippo pathway related genes from tumor tissue and paired normal tissue of gastric cancer patients in GSE29272. The blue line represents normal tissue and red represents tumor tissue. **N** Relationships of consensus module eignegenes and the expression level of OTUB1. The module name is indicated on the left side of each cell. The correlation coefficient between OTUB1 expression and module is shown in each cell, with the *p* values printed below the correlations coefficient in parentheses. Genes in each module is indicated in the corresponding cell. Red colors represent positive correlations, while blue colors denote negative correlations. IOD integrated optic density, ES enrichment score, NES normalized enrichment score.

In order to find the critical DUBs in modulation Hippo signaling, we utilized one DUB1 siRNA library, containing 100 siRNAs for each DUBs, to screen important hits. Since HEK293 cells were widely used in Hippo study and easily transfected, we utilized HEK293 as the model. CTGF, as the most classical Hippo target gene, was used as the readout (Fig. 1A). Several reported DUBs, such as USP9X and UBTD1, were identified in our screening assay, while we also identified several un-reported DUBs, which also dramatically affected CTGF expression, including OTUB1 (Fig. 1B). We further analyzed the expression of OTUB1 in gastric cancer, while the TCGA database showed that OTUB1 was higher expression in gastric cancer compared with normal gastric tissues (Fig. 1C). This conclusion was confirmed from immunohistochemistry results of 20 human gastric cancer samples (Fig. 1D, E). Further analysis revealed that OTUB1 was elevated in all stages of gastric cancer compared with normal gastric tissue (Fig. 1F). We further analyzed the prognosis of OTUB1 in gastric cancer patients from KMPLOT database (https://kmplot.com/analysis/). In the KMPLOT analysis, we found that both OTUB1 and YAP expression related to poor survival in gastric cancer patients (Fig. 1G, H). We further depleted OTUB1 in AGS gastric cancer cells for RNA sequence analysis. The GSEA (Gene Enrichment Signature Analysis) showed that the Hippo signature genes were globally decreased in OTUB1 depletion condition (Fig. 1I). The volcano plot showed that several classical Hippo target genes were decreased including CTGF, ANKRD1 and CCND1 (Fig. 1J). The KEGG pathway analysis showed that OTUB1 depletion could affect several oncogenic pathways except Hippo pathway, such as TNF pathway, TGF-b pathway and P53 signaling (Fig. 1K). The heat-map from OTUB1 depletion showed a group of Hippo target genes were inhibited under siOTUB1 conditions in AGS cells (Fig. 1L). Finally, by analyzing OUTB1 expression and Hippo signaling target genes from GSE29272, we observed that they had higher expression levels in gastric tumor compared with those in normal tissue (Fig. 1M). In addition, WGCNA analysis based on the expression prolife of GSE29272 indicated that OTUB1 was positively correlated with genes belongs to the grey module in gastric cancer (Fig. 1N). Taking together, we propose that OTUB1 could a positive regulator for Hippo/YAP signaling in gastric cancer.

### OTUB1 regulates Hippo/YAP axis in human gastric cancer cells

**Fig. 2:**
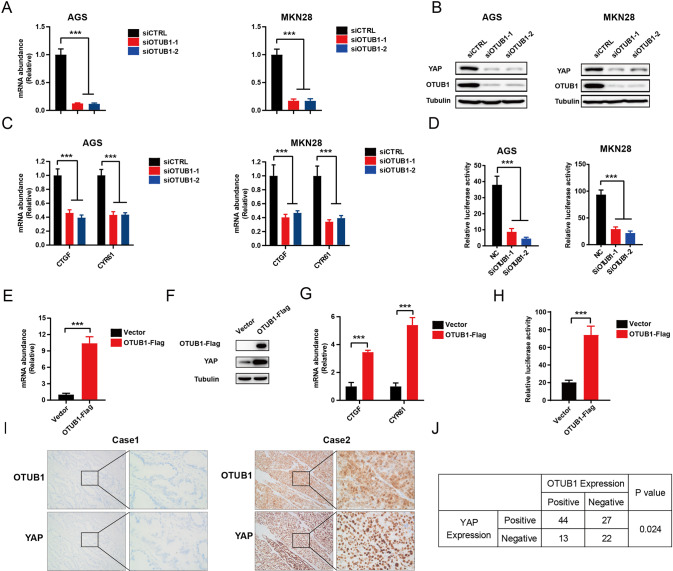
OTUB1 positively regulate Hippo pathway in gastric cancer cell lines. **A** RT–qPCR results of OTUB1 mRNA expression in AGS (left panel) or MKN28 (right panel) cell line transfected with scrambled or two independent siRNA targeting OTUB1 for 48 h, respectively. **B** Western blot results showing the expression level of YAP and OTUB1 in AGS (left panel) or MKN28 (right panel) cell line transfected with scrambled or two independent siRNA targeting OTUB1 for 48 h, respectively. **C** RT–qPCR results of CTGF and CYR61 mRNA expression in AGS (left panel) or MKN28 (right panel) cell line transfected with scrambled or two independent siRNA targeting OTUB1 for 48 h, respectively. **D** Transcriptional activity of TEAD measured by luciferase assay with a reporter containing tandem TEAD-binding sites in AGS (left panel) or MKN28 (right panel) cell line transfected with scrambled or two independent siRNA targeting OTUB1 for 48 h, respectively. **E** RT–qPCR results of OTUB1 mRNA expression in MKN28 cell line stable expressing OTUB1-flag or lentiviral vector. **F** Western blot results showing the expression level of YAP and OTUB1 in MKN28 cell line stable expressing OTUB1-flag or lentiviral vector. **G** RT–qPCR results of CTGF and CYR61 mRNA expression in MKN28 cell line stable expressing OTUB1-flag or lentiviral vector. **H** Transcriptional activity of TEAD measured by luciferase assay with a reporter containing tandem TEAD-binding sites in MKN28 cell line stable expressing OTUB1-flag or lentiviral vector. **I** Immunohistochemistry (IHC) detecting OTUB1 and YAP expression in gastric cancer specimen. **J** Chi-square test to determine the correlation between OTUB1 and YAP. Data are normalized to the values of the control group and are presented as mean ± SEM. One-way ANOVA is conducted to detect significant differences in panel **A**, **C**, **D**. The *t*-test is conducted to detect significant differences in panel **E**, **G**, **H** (^*^P < 0.05, ^**^P < 0.01, ^***^P < 0.001).

We further utilized AGS and MKN28 cell lines to explore the OTUB1 effect in Hippo signaling. QPCR assay showed that OTUB1 could be effective silenced via two independent siRNAs (Fig. 2A). The immuno-blotting data showed that OTUB1 depletion could decrease YAP protein level in AGS and MKN28 cells (Fig. 2B). Knockdown of OTUB1 doesn’t affect the expression levels of upstream components of the Hippo pathway in MKN28 cells, such as MST1, LAST1, TEAD1, TEAD2 and TAZ (Supplementary Fig. 1A). We further analyzed the Hippo target gene expression in OTUB1 depleted conditions, in which OTUB1 silencing could inhibit Hippo target gene expression in AGS and MKN28 cells, including CTGF and CYR61 (Fig. 2C). The luciferase reporter assay showed that OTUB1 inhibition could inhibit TEAD response elements activity in AGS and MKN28 cells (Fig. 2D). We further over-expressed OTUB1 in MKN28 cell via lenti-virus system. We firstly confirmed that OTUB1 were over-expressed with high efficiency (Fig. 2E). The western blot data showed that OTUB1 over-expression could increase YAP protein level in MKN28 cells (Fig. 2F). While the QPCR data showed that OTUB1 over-expression could enhance Hippo target gene expression, including CTGF and CYR61 (Fig. 2G). The luciferase reporter assay showed that OTUB1 over-expression could enhance TEAD response elements activity in MKN28 cells (Fig. 2H). We further analyzed the expression of YAP and OTUB1 in gastric cancer samples. From the immunohistochemistry analysis of 106 samples, we observed that the expression of OTUB1 was positively correlated with YAP protein (*P* = 0.024; Fig. 2I, J).

### Knockdown of YAP suppressed oncogenic phenotypes induced by OTUB1 overexpression

In order to investigate whether oncogenic phenotypes induced by OTUB1 overexpression was through increasing YAP, we carried further rescue experiments. We overexpressed OTUB1 in MKN28 cell, which was followed by further YAP knockdown. The western blot assay showed that the increased YAP protein level by OTUB1 overexpression could be rescued by YAP knockdown (Supplementary Fig. 2A). The CCK-8 assay showed that OTUB1 overexpression promoted gastric cancer growth, while YAP knockdown could rescue such growth promotion (Supplementary Fig. 2B). In the clone formation assay, the increased clone numbers caused by OTUB1 overexpression could be rescued by YAP knockdown (Supplementary Fig. 2C). The EdU staining assay implicated that YAP knockdown could partially rescue the number of EdU positive cells, which was increased by OTUB1 overexpression (Supplementary Fig. 2D). Besides, the cell cycle analysis showed that OTUB1 overexpression could induce G1 phase arrest, which could be rescued by further YAP knockdown in MKN28 cells (Supplementary Fig. 2E, F). The trans-well assay showed that the increased invasion capacity caused by OTUB1 overexpression could be recovered by YAP knockdown in MKN28 cells (Supplementary Fig. 2G). In the wound-healing assay, YAP knockdown could also recover the cell migration speed, which was increased by OTUB1 overexpression in MKN28 cells (Supplementary Fig. 2H). The PI/Annexin V double staining coupled with FACS analysis showed that overexpression of OTUB1 decreased cell apoptosis, which could be partially rescued by further YAP knockdown in MKN28 cells (Supplementary Fig. 2I, J). All these data showed that oncogenic phenotypes induced by OTUB1 overexpression could be rescued by YAP inhibition.

### OTUB1 is required for gastric cancer cell progression

**Fig. 3:**
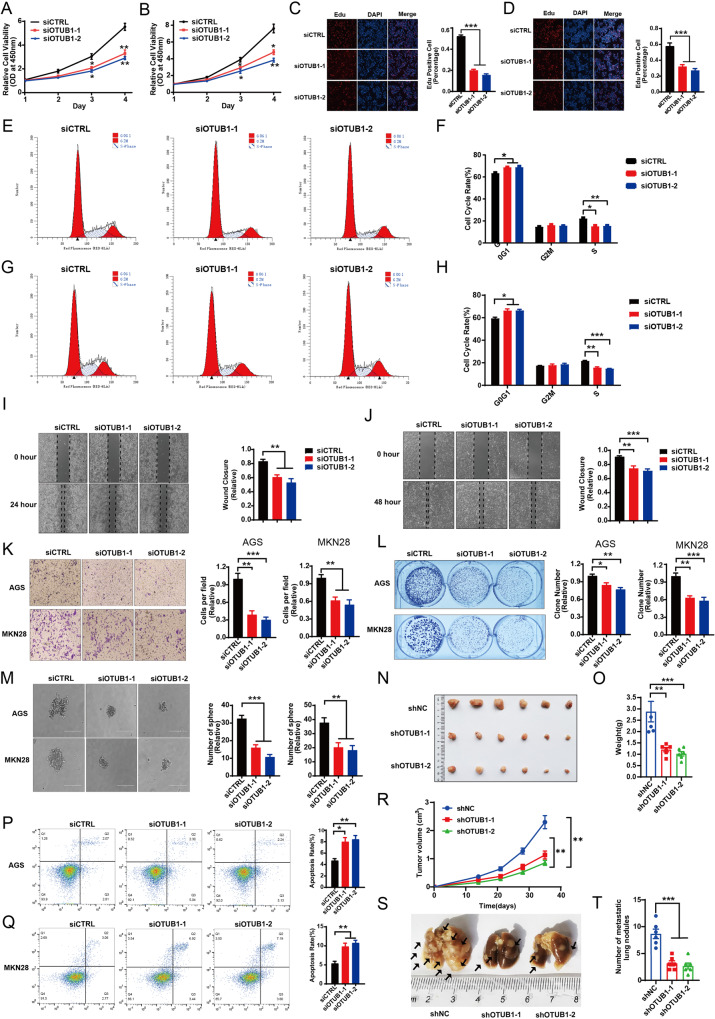
OTUB1 is required for gastric cancer cell progression. **A** Cell viability was determined by CCK8 assay in the AGS cell line transfected with either scrambled (si-CTRL) or two independent OTUB1 siRNA (si-OTUB1). **B** Cell viability was determined by CCK8 assay in the MKN28 cell lines transfected with either scrambled (si-CTRL) or two independent OTUB1 siRNA (si-OTUB1). **C** EdU assay (left panel) to show the cell proliferation of AGS cell line transfected with either scrambled (si-CTRL) or two independent OTUB1 siRNA (si-OTUB1). Right panel shows quantification of EdU results. **D** EdU assay to show the cell proliferation of MKN28 cell line transfected with either scrambled (si-CTRL) or two independent OTUB1 siRNA (si-OTUB1). Right panel shows quantification of EdU results. **E** Representative cell-cycle analysis by flow cytometry of AGS cells transfected with either scrambled (si-CTRL) or two independent OTUB1 siRNA (si-OTUB1). **F** The histogram shows quantification of cell-cycle results in AGS cells. **G** Representative cell-cycle analysis (left panel) by flow cytometry of KMN28 cells transfected with either scrambled (si-CTRL) or two independent OTUB1 siRNA (si-OTUB1). **H** The histogram shows quantification of cell-cycle results in KMN28 cells. **I** Wound healing assay (left panel) of AGS cell migration capability following transfected with either scrambled (si-CTRL) or two independent OTUB1 siRNA (si-OTUB1). Right panel shows quantification of wound healing results. **J** Wound healing assay (left panel) of KMN28 cell migration capability following transfected with either scrambled (si-CTRL) or two independent OTUB1 siRNA (si-OTUB1). Right panel shows quantification of wound healing results. **K** Transwell assay (left panel) of AGS and MKN28 cell transfected with either scrambled (si-CTRL) or two independent OTUB1 siRNA (si-OTUB1), respectively. Right panel shows quantification of transwell assay results. **L** Colony formation (left panel) of AGS cells or MKN28 cells transfected with either scrambled (si-CTRL) or two independent OTUB1 siRNA (si-OTUB1), respectively. Right panel shows quantification of colony formation assay results. **M** Spheroid formation in 3D culture AGS and MKN28 cell transfected with either scrambled (si-CTRL) or two independent OTUB1 siRNA (si-OTUB1), respectively. Left panel shows microscopic images of spheroids, and right panel shows the quantification of spheroids. **N** Representative image of tumor derived from NSG mice injected with control or OTUB1-depleted MKN28 cells is followed as indicated. **O** Quantitative tumor weight of tumors was shown. **P** Representative plots (left panel) of apoptosis ASG cell transfected with either scrambled (si-CTRL) or two independent OTUB1 siRNA (si-OTUB1), respectively. Quantitative summary (right panel) of apoptosis analysis of FACS. **Q** Quantitative tumor volumes from NSG mice injected with control or OTUB1-depleted MKN28 cells are measured at indicated time points. **R** Representative plots (left panel) of apoptosis MKN28 cell transfected with either scrambled (si-CTRL) or two independent OTUB1 siRNA (si-OTUB1), respectively. Quantitative summary (right panel) of apoptosis analysis of FACS. **S** Representative image of In vivo lung metastasis of indicated MKN28 cells were indicated. **T** Quantitative analysis of tumor metastasis in (**S**). All data are shown as mean ± SEM. *p < 0.05, **p < 0.01, ***p < 0.001 by one-way ANOVA.

We further explored the effect of OTUB1 in gastric cancer phenotypes. The CCK8 assay showed that OTUB1 depletion inhibited cancer cell proliferation in AGS and MKN28 cells (Fig. 3A, B). The EdU incorporation assay demonstrated that OTUB1 silencing dramatically reduced the EdU positive cells in AGS and MKN28 cells (Fig. 3C, D). The FACS analysis showed that OTUB1 depletion could cause G1 cell cycle arrest in both AGS and MKN28 cells (Fig. 3E–H). While in the wound-healing assay, we found that OTUB1 depletion inhibited wound closure speed in both AGS and MKN28 cells (Fig. 3I, J). The trans-well assay showed that OTUB1 knocking-down inhibited cell invasion capacity in AGS and MKN28 cells (Fig. 3K), while the clone formation assay showed that OTUB1 depletion could significantly reduce the numbers of clones in AGS and MKN28 cells (Fig. 3L). The 3D culture assay indicated that OTUB1 was required for the sphere formation in AGS and MKN28 cells (Fig. 3M). We further investigated the effect of OTUB1 in vivo. The xenograft model showed that OTUB1 depletion significantly reduced tumor growth in vivo (Fig. 3N, O, R). We further investigated the effect of OTUB1 in cell death. The Propidium Iodide (PI) coupled with Annexin V staining showed that OTUB1 depletion facilitated cancer cell apoptosis in AGS and MKN28 cells (Fig. 3P, Q). Finally, our carried out the in vivo metastasis assay, in which OTUB1 depletion could reduce lung metastasis capacity in MKN28 cells (Fig. 3S, T). All these data indicate OTUB1 plays important roles in facilitating gastric cancer progression.

### OTUB1 promotes gastric cancer progression via Hippo/YAP axis

**Fig. 4:**
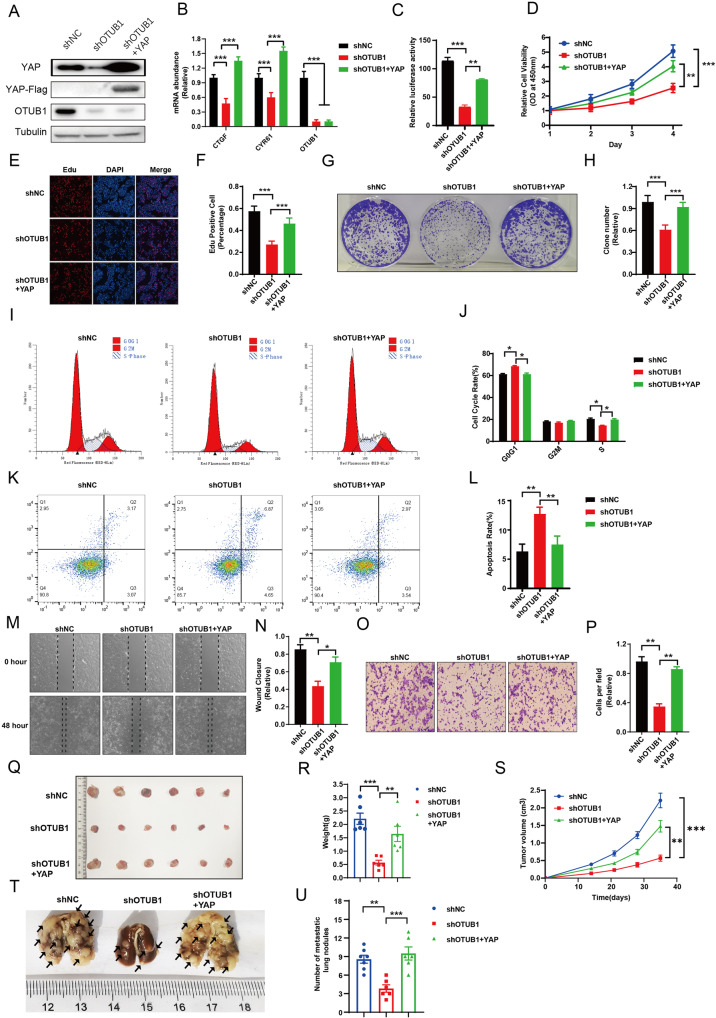
YAP rescues the defects of gastric cancer cells induced by OTUB1 depletion. **A**–**P** MKN28 cells were transduced with lentiviruses expressing control or shRNA targeting OTUB1, following with YAP overexpression. **A** MKN28 cells were subjected to immunoblotting as indicated. Flag was fused to YAP. Tubulin served as loading control. **B** Relative RNA level of CTGF and CYR61 in indicated MKN28 cells was measured. **C** Relative YAP-TEAD luciferase activity was measured in MKN28 cells as indicated. **D** Cell growth of MKN28 cells were measured by CCK-8 assay. **E**, **F** Cell proliferation of indicated MKN28 cells were measured by EdU staining. **E** Representative image of EdU staining. **F** Quantitative analysis of EdU staining in **E**. **G**, **H** Clone formation of MKN28 cells were measured as indicated. **G** Representative image to show crystal violet staining of clones. **H** Quantitative summary of clones in **G**. **I**, **J** Cell cycle of indicated MKN28 cells were measured by FACs. **I** Representative cell cycle plots by FACs. **J** Quantitative summary of cell cycle analysis in (**I**). **K**, **L** Apoptosis of indicated MKN28 cells were measured by FACs. **K** Representative plots of cell apoptosis by FACs. **L** Quantitative summary of apoptosis analysis in **K**. **M**, **N** Cell migration of MKN28 cells was measured by wound-healing assay as indicated. **M** Representative cell image of wound-healing assay. **N** Quantitative summary of wound-healing assay in **M**. **O**, **P** Cell invasion of indicated MKN28 cells was measured by trans-well assay. **O** Representative cell image of trans-well assay. **N** Quantitative summary of trans-well assay in **O**. **Q**–**U** MKN28 cells were transduced with lentiviruses expressing control or shRNA targeting OTUB1, following with YAP overexpression. **Q**–**S** Control, OTUB1-depleted or OTUB1-depleted with ectopic YAP expressed MKN28 cells were injected into NSG mice and tumor growth was followed as indicated. Representative image(**Q**) and quantitative tumor weight (**R**) were shown. Quantitative tumor volumes were measured at indicated time points. **T**, **U** In vivo lung metastasis of indicated MKN28 cells were measured. **T** Representative image to show the lung metastasis of tumors. **U** Quantitative analysis of tumor metastasis in **T**. All data are shown as mean ± SEM. *p < 0.05, **p < 0.01, ***p < 0.001 by one-way ANOVA.

We carried further rescue experiments to test if OTUB1 modulated cancer progression through YAP. The western blot assay showed that the reduced YAP protein level by OTUB1 depletion could be rescued by YAP over-expression (Fig. 4A). The QPCR data and luciferase assay showed that YAP over-expression could rescue Hippo target gene activity, which was reduced by OTUB1 silence (Fig. 4B, C). The CCK-8 assay and EdU staining assay showed that OTUB1 depletion inhibited gastric cancer growth, while YAP over-expression could partially rescue such growth inhibition (Fig. 4D–F). In the clone formation assay, the reduced clone numbers caused by OTUB1 depletion could be partially rescued by YAP over-expression (Fig. 4G, H). Besides, the cell cycle analysis showed that OTUB1 depletion could induce G1 phase arrest, which could be partially rescued by further YAP over-expression in MKN28 cells (Fig. 4I, J). The PI/Annexin V double staining coupled with FACS analysis showed that the knockdown of OTUB1 increased cell apoptosis, which could be partially rescued by further YAP over-expression in MKN28 cells (Fig. 4K, L). In the wound-healing and trans-well assays, YAP over-expression could also recover the cell migration and invasion speed, which was reduced by OTUB1 depletion in MKN28 cells (Fig. 4M, P). In the xenograft mice model, OTUB1 depletion inhibited tumor growth, which effect could be rescued by YAP over-expression in MKN28 cells (Fig. 4Q, S). In the in vivo metastasis assay, OTUB1 depletion could significantly reduce the lung metastasis, which effect could be partially rescued by YAP over-expression in MKN28 cells (Fig. 4T, U).

As YAP-S5A is a constitutively active YAP mutant, we further test whether the phenotype induced by OTUB1 knockdown could be rescued by YAP-S5A. We knockdowned the OTUB1 expression by lentiviral vector system in MKN28 cell, which was followed by further YAP-S5A overexpression. The western blot assay showed that the reduced YAP protein level by OTUB1 depletion could be covered by YAP-S5A over-expression (Supplementary Fig. 3A). The CCK-8 assay showed that OTUB1 depletion inhibited gastric cancer growth, while YAP-S5A over-expression could totally rescue such growth inhibition (Supplementary Fig. 3B). The clone formation assay showed that the reduced clone numbers caused by OTUB1 depletion could be completely rescued by YAP-S5A over-expression (Supplementary Fig. 3C). The EdU staining assay implicated that YAP-S5A over-expression could rescue the decreased amount of EdU positive cells, which was induced by OTUB1 knocking-down (Supplementary Fig. 3D). Furthermore, the cell cycle analysis showed that OTUB1 depletion could induce G1 phase arrest, which could be partially rescued by further YAP-S5A over-expression in MKN28 cells (Supplementary Fig. 3E, F). The trans-well assay showed that the impaired invasion capacity caused by OTUB1 depletion could be partially recovered by YAP-S5A over-expression in MKN28 cells (Supplementary Fig. 3G). The wound-healing assay indicated that YAP-S5A over-expression could also recover the cell migration speed, which was reduced by OTUB1 depletion in MKN28 cells (Supplementary Fig. 3H). The PI/Annexin V double staining coupled with FACS analysis showed that knockdown of OTUB1 increased cell apoptosis, which could be rescued by further YAP-S5A over-expression in MKN28 cells (Supplementary Fig. 3K, L). These results indicated that the phenotype induced by OTUB1 knockdown could be rescued by YAP-S5A.

### OTUB1 stabilizes YAP protein via inhibiting K48-linked polyubiquitination of YAP at several lysine sites

**Fig. 5:**
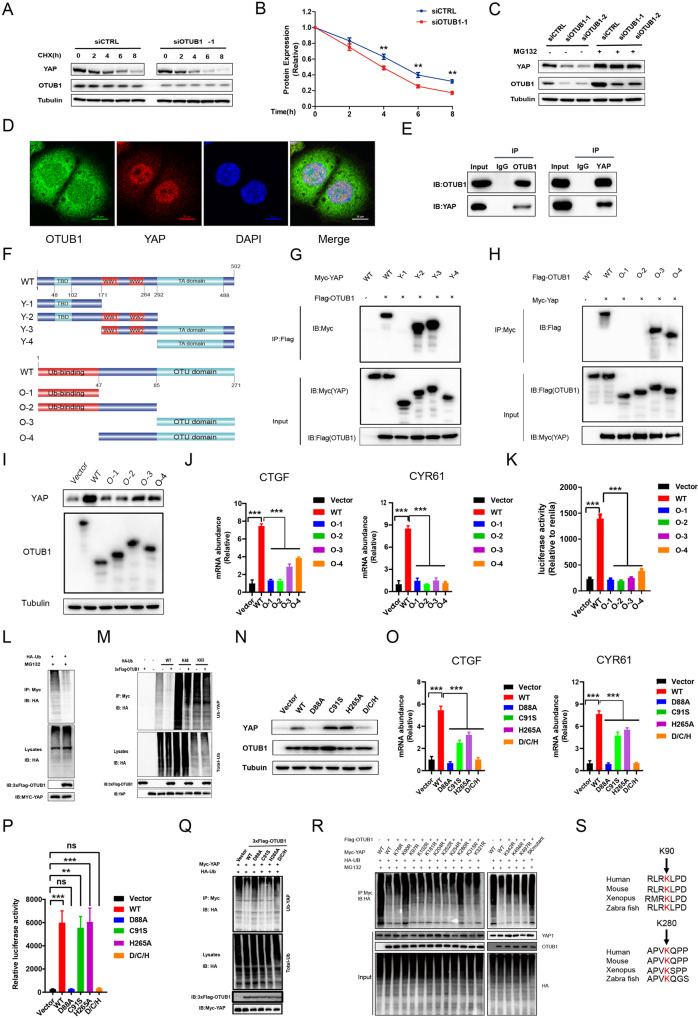
OTUB1 stabilizes YAP protein in gastric cancer cells. **A**, **B** Control or OTUB1-slienced MKN28 cells were treated with cycloheximide (CHX) and cells were collected at indicated time point upon treatment. **A** Representative immunoblot of YAP and OTUB1 was shown. Tubulin served as loading control. **B** Quantitative analysis of halftime of YAP protein in **A**. **C** Control or OTUB1-slienced MKN28 cells were treated with MG132. YAP and OTUB1 protein were measured by immunoblotting. Tubulin served as loading control. **D** Immunofluorescence imaging of OTUB1(Green), YAP(Red) and DAPI (blue)in MKN28cells. **E** Representative immunoblots to show the interaction between OTUB1 and YAP as assessed by immunoprecipitation (IP) for OTUB1 or YAP, compared to isotype control (IgG) (10% input). **F** Schematic diagram to show wild-type and truncated OTUB1 and YAP constructs used in this study. **G** Representative immunoblots to show the interaction between OTUB-1 and WT or truncated YAP as indicated assessed by immunoprecipitation (IP) with OTUB1 (Flag). **H** Representative immunoblots to show the interaction between YAP and WT or truncated OTUB-1 as indicated assessed by immunoprecipitation (IP) with YAP (anti-Myc). **I** MKN28 cells were transfected with WT and truncated OTUB-1 and then subjected to immunoblotting to examine YAP protein level. **J** Relative RNA level of CTGF and CYR61 in MKN28 cells transfected with control vector, WT and truncated OTUB1 constructs. **K** Relative YAP-TEAD luciferase activity was measured in MKN28 transfected with control vector, WT and truncated OTUB1 constructs. **L**, **M** HEK293 cells were co-transfected with YAP and control or OTUB1 constructs as indicated. Global ubiquitination (**L**), K48- and K63-linked ubiquitination of YAP(**M**) were measured by ubiquitination assay. **N** WT and four OTUB1 mutants (D88A, C91S, H265A, D/C/H triple mutants) were transfected into HEK293 cells. Cells were subjected to immunoblotting to examine YAP protein level. Tubulin served as loading control. **O** Relative RNA level of CTGF and CYR61 were measured in MKN28 cells transfected with WT or OTUB1 mutants. **P** Relative YAP-TEAD luciferase activity were measured in MKN28 cells transfected with WT or OTUB1 mutants. **Q** Global ubiquitination of YAP in MKN28 cells transfected with WT or OTUB1 mutants was measured by ubiquitination assay. **R** Ubiquitination of YAP at indicated multiple sites were measured by ubiquitination assay. **S** Conservative analysis of K90 and K280 on YAP protein was performed across multiple species. All data are shown as mean ± SEM. *p < 0.05, **p < 0.01, ***p < 0.001 by one-way ANOVA.

Since OTUB1 modulates Hippo/YAP axis and gastric cancer progression, we further investigated the potential mechanism. The protein half-life assay showed that OTUB1 depletion impaired the YAP protein stability in gastric cancer cells (Fig. 5A, B). Since YAP degradation relied on the effect of proteasome, we utilized the proteasome inhibitor MG132. OTUB1 depletion could decrease YAP protein level, while the MG132 treatment could diminish such decrease in YAP protein (Fig. 5C). The immuno-staining showed that YAP protein was mainly located in the nuclear, while OTUB1 located both in the cytosol and nuclear (Fig. 5D). The endogenous immuno-precipitation assay indicated that OTUB1 could associate with YAP in gastric cancer cells (Fig. 5E). We further investigated the interaction domains between OTUB1 and YAP. YAP protein is composed of TB (TEAD binding domain), WW domain and TA domain (Trans-activation domain), while the OTUB1 protein is composed of the ubiquitin-binding domain at the N-terminus and the OTU domain at the C-terminus (Fig. 5F). We made these deletion constructs for interaction analysis, which revealed that the WW domain was required for YAP to interact with OTUB1 while the OTU domain was necessary for OTUB1 to associate with YAP (Fig. 5G, H). We further over-expressed the deletion domains of OTUB1 to check their impact on Hippo/YAP axis. The western blot data showed that only full length of OTUB1 could stabilize YAP protein (Fig. 5I). The QPCR data showed that the intact OTUB1 was required to increase endogenous Hippo target gene expression and TEAD luciferase activity (Fig. 5J, K).

Since OTUB1 belongs to the DUB family, we further checked the effect of OTUB1 on YAP ubiquitination. The ubiquitination assay showed that OTUB1 over-expression could significantly decrease YAP total ubiquitination level and K48-linked ubiquitination, but little effect on K63-linked ubiquitination (Fig. 5L, M). Since previous studies showed that the catalytic center of OTUB1 harbored a triad, including aspartate 88, cysteine 91 and histidine 265, we made these mutation variants and transfected into HEK293 cells. The western blot data indicated D88 site of OTUB1 was required for YAP protein level (Fig. 5N). The QPCR data and luciferase data implicated that D88A mutant form abolished OTUB1 effect on Hippo target gene expression and TEAD luciferase activity (Fig. 5O, P). Further ubiquitination assay showed that D88A mutation variants could not inhibit YAP poly-ubiquitination as OTUB1 WT form did (Fig. 5Q). These assays showed that the site aspartate 88 is critical for OTUB1 to exert its deubiquitination effect on YAP. We further examined the detailed ubiquitination sites on YAP protein. Since YAP protein contains 14 lysine sites, which could be ligated, we made these lysine mutant forms. The ubiquitination assay showed that OTUB1 could inhibit YAP protein poly-ubiquitination at several sites, including K90, K280, K342, K494 and K497 (Fig. 5R). Among these sites, the K90 and K280 sites are evolutionally conserved from zebra fish to human (Fig. 5S). Additionally, In vitro ubiquitination assay indicated that OTUB1 could promote YAP deubiquitination In Vitro (Supplementary Fig. 4A–D). Moreover, His-tagged YAP could directly interacted with GST-fused OTUB1 in an in vitro pull-down assay (Supplementary Fig. 4E).

## Discussion

**Fig. 6:**
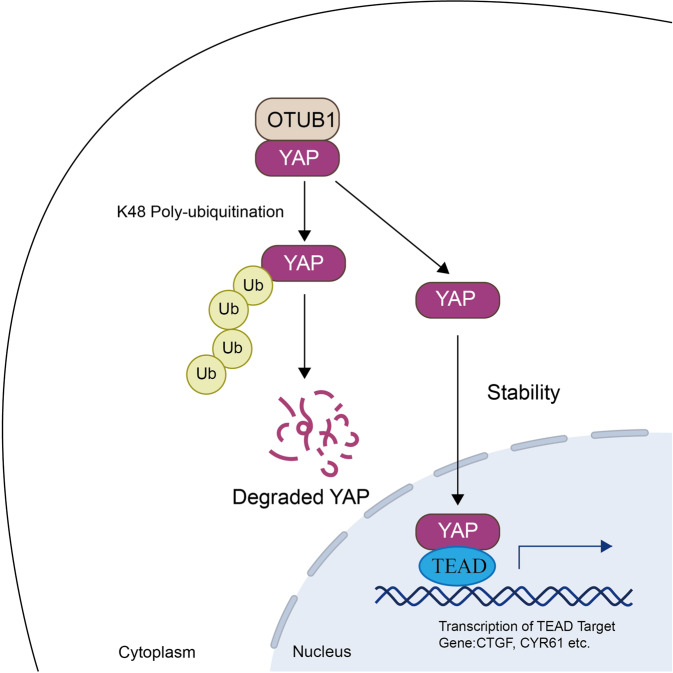
Hypothetical model for the functional interplay of OTUB1 with Hippo signaling in gastric cancer cells. OTUB1 associates with YAP, promotes YAP K48-linked ubiquitination and degradation in gastric cancer cells, which promotes the activation of Hippo/YAP axis and gastric cancer progression.

In the study, we identified OTUB1 as a critical factor for gastric cancer progression. OTUB1 associated with YAP and modulated YAP protein activity and stability, which subsequently exerted its critical decisions on Hippo on or off status (Fig. 6). Since OTUB1 has been proved as an important deubiquitinase in Hippo signaling, targeting OTUB1 function or modulating OTUB1 expression could be a plausible strategy for gastric cancer therapeutics.

According to recent world cancer statistics, there are more than one million new cases of gastric cancer diagnosed worldwide, while 60% of them are found in China [19, 20]. The overall incidence and mortality of gastric cancer rank top 3 among all malignancies in China. The 5-year overall survival of gastric cancer is around 35%, while most of the cases are diagnosed at advanced stages [21]. Thus, systematic treatments, including medical treatments, are the major therapies for advanced gastric cancer [22]. Along with the advance of basic research in gastric cancer, several novel strategies have been achieved, such as Herceptin for HER2 expression patients and PD1 targeting immuno-therapy [23, 24]. However, the 5-FU based chemotherapy is still the major choice for most of advanced gastric cancer patients [25]. Although molecular classification of gastric cancer has been set up, which includes EBV (Espstein-Barr virus) positive type, MSI type (Microsatellite Instability), GS type (Genomic Stable) and CIN type (Chromosomal Instability), the correspondent targeting therapy for each subtype is still premature [26–28]. Thus, it is of great importance to identify novel therapeutic targets for gastric cancer treatments.

The Hippo signaling is an auto-inhibition pathway, which controls the tissue regeneration and organ size in physiological conditions. When Hippo pathway is activated, the phosphorylation kinase cascade from MST1/2 to LATS1/2 facilitates YAP phosphorylation, which retains YAP in the cytosol for degradation. However, if Hippo/YAP axis is over-activated, YAP could trans-locate into the nuclear and facilitate LATS2 transcription. As one of the YAP target genes, increased LATS2 expression could further restrain YAP function and Hippo/YAP activation. Thus, the Hippo signaling could maintain such delicate balance, which is relied on the functional phosphorylation cascade. In human cancers, there were several studies reporting that the dysfunction of Hippo pathway inhibitors, including FATs mutations, could lead to the over-activation of Hippo/YAP axis [29]. However, in gastric cancer, such mutations of Hippo inhibitors were rarely reported. It is still a mystery that why Hippo/YAP axis is over-activated, while the auto-inhibition cascade remains functional. Recently studies reveal that the other forms of post-translational modifications, such as ubiquitination, are also important in preventing YAP over-activation. For example, our recent study revealed a few E3 ubiquitin ligases could associate with YAP protein and facilitate its proteasome dependent degradation.

Since YAP protein could be ubiquitinated by several E3 ubiquitin ligases, there is no doubt the ubiquitination level of YAP is important in regulation Hippo signaling activity and gastric cancer cell progression. Inhibition of critical deubiquitinases function or their expression, which could enhance YAP poly-ubiquitination and degradation, could be a promising strategy to block Hippo/YAP axis function and restrain gastric cancer progression. Several researchers made effort to identify critical deubiquitinases in Hippo pathway in other cancers via deubiquitinase siRNA library [30]. Our DUBs siRNA screening data not only provided an overall picture of DUBs effects on Hippo signaling, but also identified OTUB1 as a critical deubiquitinase in modulating YAP stability and gastric cancer progression. OTUB1 was firstly discovered as a Yersinia protein kinase substrate in 2006 [31]. Further studies revealed that OTUB1 belonged to OTU domain family deubiquitinase and modulated several biological processes. For example, OTUB1 could associate with P53 and stabilize P53 signaling. Besides, OTUB1 could also inhibit c-IAP degradation and enhance the signaling of NFKB and MAPK pathway [32]. In malignancies, OTUB1 was found to elevated its expression in several human cancers, which expression was also associated with poor survival in quite a few studies. For example, OTUB1 could promote cancer cell immunosuppression via stabilizing PD-L1 [18]. In addition, OTUB1 could facilitate lung cancer formation via stabilizing RAS signaling [33]. In gastric cancer, although there was report showing OTUB1 was higher expression in gastric malignancies compared with normal tissue, the detailed mechanism was not clear [34]. Interestingly, our screening data indicated OTUB1 was a novel deubiquitinase in modulating Hippo signaling function in gastric cancer. Combined with the other oncogenic role of OTUB1 in previous studies, blocking OTUB1 could result in the inhibition of multiple oncogenic pathways and synchronize in gastric cancer therapy.

In conclusion, we discovered the novel regulation link between Hippo/YAP axis and OTUB1 in gastric cancer. Our clinical sample and biological studies showed that OTUB1 was an oncogene in supporting gastric cancer proliferation and metastasis in vitro and in vivo. OTUB1 could associate with YAP and deubiquitinate YAP protein at multiple lysine sites, which subsequently lead to enhanced Hippo/YAP activity. Based on the regulation link, modulation of OTUB1 expression or function could be a promising strategy to treat YAP-driven cancers.

## Materials and methods

### Cell Culture

AGS, MKN28, and HEK293 cells are acquired from American Type Culture Collection. AGS and MKN28 cells are cultured with RPMI-1640 (Hyclone) supplemented with 2 mM L-glutamine and 10% FBS (10270, Life Technologies). HEK293 cells are cultured with Dulbecco’s Modified Eagle’s Medium that contains 4, 5 g/L glucose and 4 mM L-glutamine (Life Technologies) supplemented with 10% FBS.

### Plasmids and siRNA

The expression plasmid encoding OTUB1-flag and OTUB1 truncation mutants were constructed by PCR and subsequent insertion of the corresponding fragment into GFP-N1 vector. The YAP-myc and YAP truncation mutants were constructed by PCR and subsequent insertion of the corresponding fragment into pcDNA3.1 vector. The HA-K48, K63, HA-Ub and TEAD-reporter plasmids were purchased from Addgene. The plvx-shRNA2-OTUB1, pCDH-EF1-OTUB1-flag, pCDH-EF1-YAP-flag and pCDH-EF1-YAP-S5A-flag were constructed into corresponding vector. To produce lentivirus, the expression vector transfected into HEK293 cells along with the packaging plasmid psPAX2 and the envelope plasmid pMD2.g using Lipofectamine 2000 (Invitrogen). The siRNA sequences were as follows: siOTUB1-1: 5′-GAC GGC AAC UGU UUC UAU C-3′, and siOTUB1-2: 5′-GAC GGA CUG UCA AGG AGU U-3′. The shRNA sequences were as follows: shOTUB1-1: GGA TCC GTT AAC TGT CTG GCC TAT GAT TCA AGA GAT CAT AGG CCA GAC AGT TAA TTT TTT GAA TTC and siOTUB1-2: GGA TCC GGA CGG ACT GTC AAG GAG TTT TCA AGA GAA ACT CCT TGA CAG TCC GTC TTT TTT GAA TTC.

### Luciferase reporter assay

AGS or KMN28 cells were seeded in 48-well plates. Luciferase reporter plasmid along with Renila expression plasmid and the indicated plasmids were co-transfected into corresponding cells using Lipo 2000. After 48 h, post-transfection, cells were lysed for Hippo signaling activity and the activities of firefly luciferase and renilla luciferase were measured using the Dual-Luciferase Reporter Assay System (Promega).

### RNA Extraction and real-time PCR

Total RNA was isolated using Trizol reagent (Invitrogen) and then reverse-transcribed into first strand cDNA using HiScript II Q Select RT SuperMix (Vazyme, #R233-01). cDNAs were subjected to quantitative real-time PCR with gene-specific primers on Step-one Plus Real-Time PCR System (Applied Biosystems) using SYBR-Green PCR mix (CWbio, #CW0659). 36B4 served as internal control. Primers used were were as follows: *OTUB1*: 5′-GTC TGC CAA GAG CAA GGA AG-3′ (forward); 5′- GCT TCT CCA CCT GCT CAA TC-3′ (reverse). CTGF: 5′-CAG CAT GGA CGT TCG TCT G-3′ (forward); 5′- AAC CAC GGT TTG GTC CTT GG-3′ (reverse). CYR61: 5′-GGT CAA AGT TAC CGG GCA GT-3′ (forward); 5′- GGA GGC ATC GAA TCC CAG C-3′ (reverse). 36B4: 5’-CGA CCT GGA AGT CCA ACT AC-3’ (forward); 5′-ATC TGC TGCA TCT GCT TG-3′ (reverse).

### Cell viability assay

AGS and MKN28 cells were transfected with si-OTUB1 or si-scrambled in 12-well plate. Twenty-Four hours after transfection, the cells number was collected and 3,000 cells were seeded into 96-well plates. The cell viability was obtained at indicated time points using the CCK8 kit (Biomake, #B34302).

### EdU assay

Cell proliferation assays were determined by using Cell Counting Kit-8 (Ribobio, # C10310-2). AGS and MKN28 cells were seeded in 24-well plates for transfection with scrambled or two independent siRNA targeting OTUB1. After 48 h, cells were added with EdU and continued incubating for another 2 h. Then, the cells were fixed with a 4% paraformaldehyde solution for 30 min. The staining process was performed according to the manufacturer’s instructions. Images were captured using an EVOS FL Auto microscope (Life Technologies) and the numbers of positive cells were calculated using the imageJ software. Results were reproduced in three biologically independent experiments.

### Wound healing assay

AGS and MKN28 cells were seeded in 6-well plate and transfected with scrambled or two independent siRNA targeting OTUB1. When cell density appeared in 100% confluence, scratching the cells with the tips of yellow pipette. The wound area was measured by ImageJ software at indicated time points and normalized with starting time point.

### Western blotting

Cells were harvested and lysed with RIPA buffer containing protease and phosphatase inhibitor cocktail (biomake, #B14001, #B15001). Proteins were loaded and separated by electrophoresis on SDS-polyacrylamide gel electrophoresis (SDS-PAGE) and transferred to a nitrocellulose membrane (Beyotime, #FFN08). Antibodies used were anti-flag antibody (diluted 1:1000, Sigma, #F1804), anti-OTUB1 antibody (diluted 1:1000, Abcam, #ab270959), anti-Tubulin antibody (diluted 1:1000, Proteintech, #Cat No. 11224-1-AP), myc antibody (1:1000, Abmart, #20002), YAP antibody (diluted 1:500, #SC101199) and anti-actin antibody (diluted 1:1000, Abclonal, #AC026). Peroxidase-Conjugated AffiniPure Goat Anti-Mouse IgG (Beyotime, #A0216) or Goat Anti-Rabbit IgG (Beyotime, #A0208). The signals were visualized using the ECL Kit (Meilunbio, #MA0186).

### Co-Immunoprecipitation assay

Each baits and prey pair plasmids were co-transfected into HEK293T cells. After 48 h culture, the transfected cells were harvested and lysed using passive Cell Lysis Buffer with protease inhibitors on ice for 30 min. The supernatant of each sample was mixed with 1 μl flag-antibody. The corresponding IgG was used as the negative control. The mixture was incubated for 4 h with gentle rotation then washed three times with 1× lysis/binding/wash buffer. After gentle rocking at 4 °C overnight, agarose-protein A/G beads (Santa Cruz Biotechnology, #sc-2003) were then added and incubated at room temperature for 4 h. The bound proteins were eluted with 2X SDS-PAGE buffer by incubating the sample for 15 min at 98 °C and then analyzed by western blotting.

### Poly-ubiquitination detection assay

To directly detect the enriched overall ubiquitinated, K63-ubiqutinated or K48-ubiqutinated YAP from the cell extracts, cells were transfected with corresponding Ub plasmid, YAP-myc together with OTUB1-flag or vector. After 48 h, total protein was extracted and pre-cleared with 50 ul protein A for overnight. The supernatant was collected and immunoprecipitated by myc antibody. Western blot was performed to detect total and ubiquitinate level of YAP.

### Immunofluorescence assay

KMN28 cells were fixed with 4% paraformaldehyde in PBS for 30 min, permeabilized with 0.1% Triton X-100 for 10 min, and blocked by 5% BSA in PBS for 1 h. A rabbit anti-OTUB1 and mouse anti-YAP antibodies were used, followed by Alexa Flour 488 anti-rabbit antibody and Alexa Flour 488 anti-mouse antibodies. Images were captured using Nikon A + laser scanning confocal system.

### RNA sequence and data analysis

Three biological replicates were prepared for the RNA-seq library and RNA-Seq analysis. For each repeat, equal amount of RNA was pooled for RNA-Seq library construction. Construction of RNA-seq library and RNA-seq was performed by BGI company (Shenzhen, China). Differential expressed genes (DEGs) are analyzed using DESeq2 packages in R language. Genes with log2(fold change) > 1 and FDR < 0.05 were considered as significant differential expressed genes. KEGG pathway enrichment analysis was performed using clusterProfiler package in R language. The RNA sequence data are deposited in the Gene Expression Omnibus (GEO) database (Assessing number: GSE197135). GSEA was performed using GSEA software (http://www.broadinstitute.org/gsea) with 1000 permutations of the gene sets.

### TCGA data download and survival analysis

Expression data of gastric cancer patients were downloaded from TCGA data portal. Kaplan–Meier survival analysis was performed using KMplot database (KMplot.com). For analyzing OUTB1 expression and Hippo signaling target genes, Expression data of gastric cancer patients were downloaded from GEO, accession number GSE29272. Volcano plot was performed using the OmicStudio tools at https://www.omicstudio.cn/tool. Heatmap plot was performed using the Morpheus tools at https://software.broadinstitute.org/morpheus/.

### 3D culture assay

MKN28 and AGS were grown in ultra-low attachment plates (Nunc, # 174931) containing RPMI1640 medium (Servicebio, #G4530) supplemented with B27 (Gibco, Grand island, NY, USA), 20 ng/ml EGF, and 20 ng/ml bFGF (Peprotech, Rocky Hill, NJ, USA). After culturing for 14 days, spheres with diameters of > 40 µm were counted.

### Cell cycle and apoptosis analysis

Cell cycle analysis was determined using propidium iodide (PI) staining. Cells (1 × 10^7^) were washed with PBS and fixed via 70% ethanol at room temperature for 30 min. After washing three times with PBS, cells were stained with PI contailing RNase A (Thermo, # F10797). Red signal was measured with a FACScan (Millipore). FSC data were analyzed using ModFIT LT v 3.1 software. For the apoptosis assay, MKN28 and AGS were staining by Annexin V and propidium iodide (PI) dual staining using an Annexin V-FITC Apoptosis Detection Kit (Vazyme, #A211-02). Fluorescence was measured with a FACScan (Millipore). FSC data were analyzed using Flowjo7.6 software.

### In vitro ubiquitination assay

YAP and FBXW7 were cloned into the pET26b vector. OTUB1 were cloned into the pGEX-4T1 vector. Protein purification followed the general procedure in the pET System Manual. Ubiquitination was performed with the ubiquitination kit (Boston Biochem) following protocols recommended by the manufacturer. Recombinant proteins were incubated with 20X E1 Enzyme, 10X Mg^2+^-ATP Solution, 10X Ubiquitin Solution, 1 ug E2 Enzyme (UbcH7, Boston Biochem; UBE2D1, Sino Biological Inc.) in a final volume of 20 µl reaction buffer at 37 °C for 1 h. Finally, products were analyzed by western-blot assays.

### Weighted correlation network analysis (WGCNA)

WGCNA approach (WGCNA R package) is performed the to recognize gene module highly correlated with the expression of OTUB1. The analysis process includes: first, downloading expression data from GEO; second, creating gene co-expression networks through calculating the connection strength; third, assigning genes into modules using hierarchical clustering with a dynamic tree cut method; fourth, construction of the relationships between gene module and the expression value of OTUB1. Finally, a scale-free topology model is built. The relationships between modules and OTUB1 expression level were analyzed with Pearson correlation coefficient and visualized by heatmap.

### Animal model

For subcutaneous injection, 5 × 10^6^ cancer cells were injected into the lower back region of 4-week-old female NSG mice, with 6 mice per group. The mice were sacrificed after 6–8 weeks. For lung metastasis model, 1 × 10^6^ single cells were injected into 4-week-old female NSG mice through the tail vein. The mice were sacrificed until one of the mice had developed severe breath burden.

### In vitro GST Pull-down assay

For pull-down assay, the GST-OTUB1 and His-YAP proteins were expressed and purified from E. coli. GST-agarose bead bound GST-OTUB1 or GST proteins were incubated with purified His-YAP. The elutes were analyzed by western blot with the indicated antibodies.

## Supplementary information

Supplementary figure 1

Supplementary figure 2

Supplementary figure 3

Supplementary figure 4

Supplementary figure legends

## Data Availability

The publicly available data are provided in GEO database. The original siRNA screening data are provided in supplementary materials. The original data for WB and QPCR are provided in supplementary materials. The cell line authentications are shown in supplementary materials. The approved document of human sample ethnics is shown in the supplementary materials.
