# Supplementary figures and images for: OTUB1 suppresses Hippo signaling via modulating YAP protein in gastric cancer
Source: Oncogene. 2022 Oct 21;41(48):5186–98. doi: 10.1038/s41388-022-02507-3 (PMC9700521; doi:10.1038/s41388-022-02507-3)

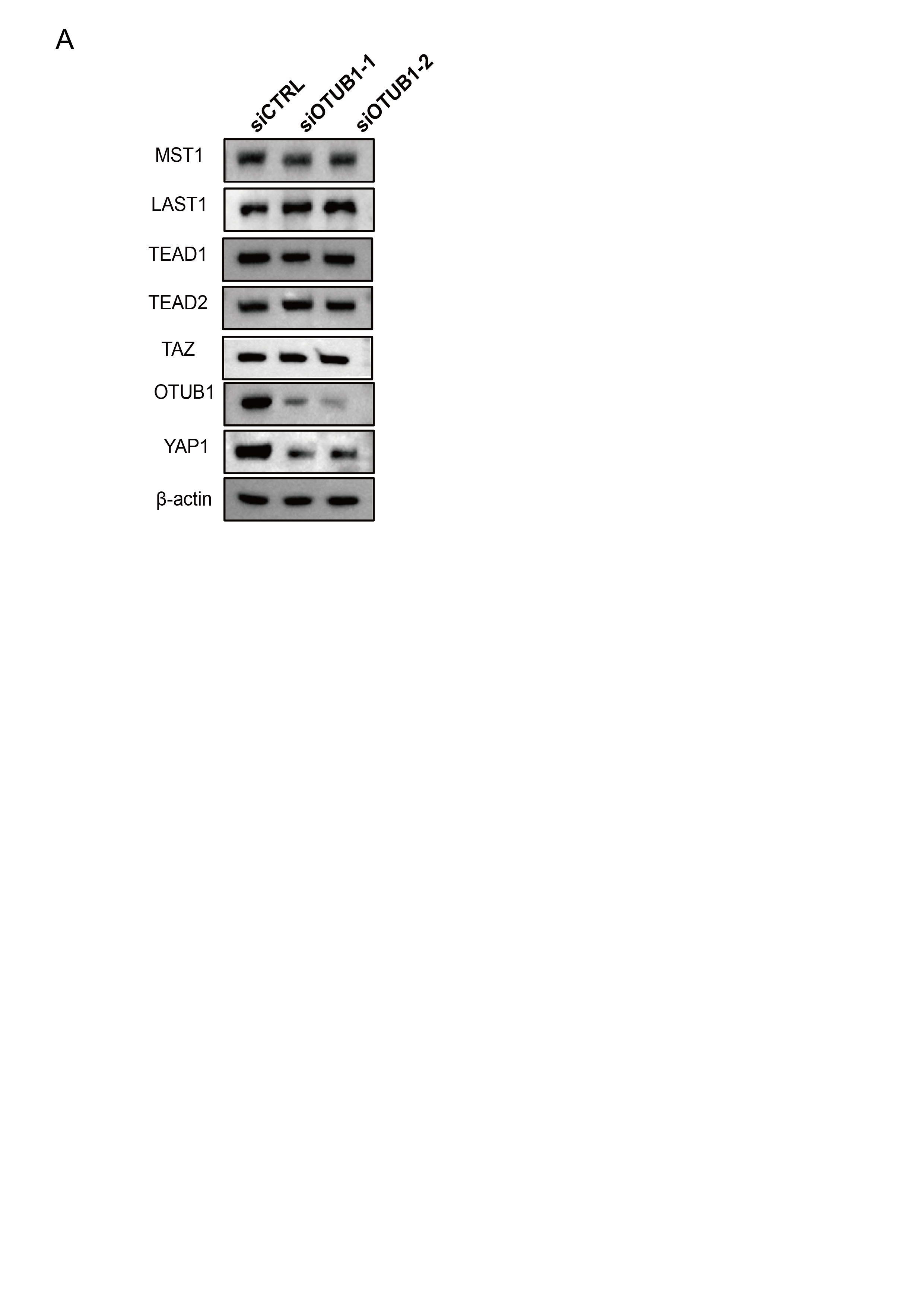

Supplement: Supplementary file 1 — Supplementary figure 1 [file 41388_2022_2507_MOESM1_ESM.jpg]

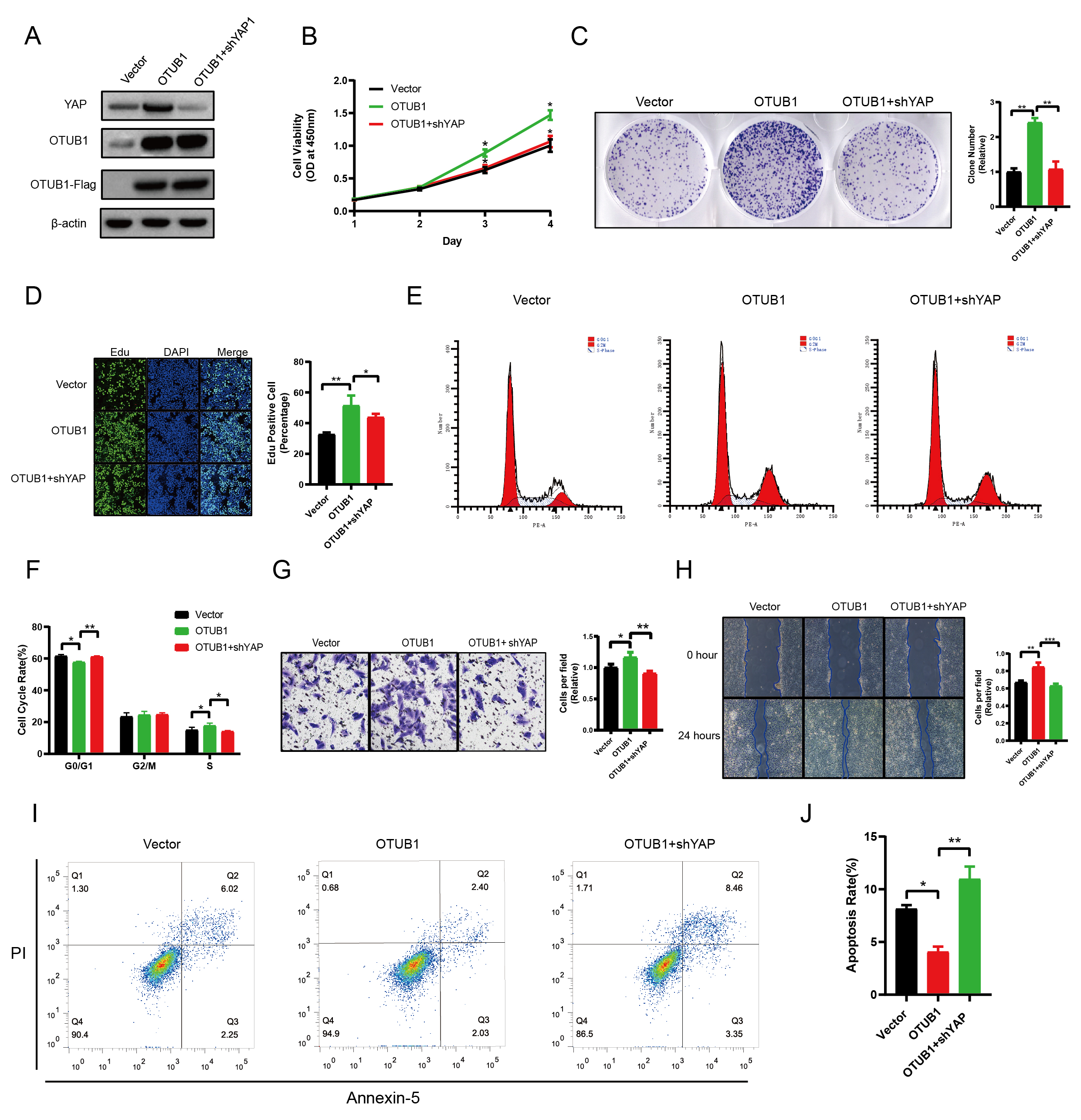

Supplement: Supplementary file 2 — Supplementary figure 2 [file 41388_2022_2507_MOESM2_ESM.jpg]

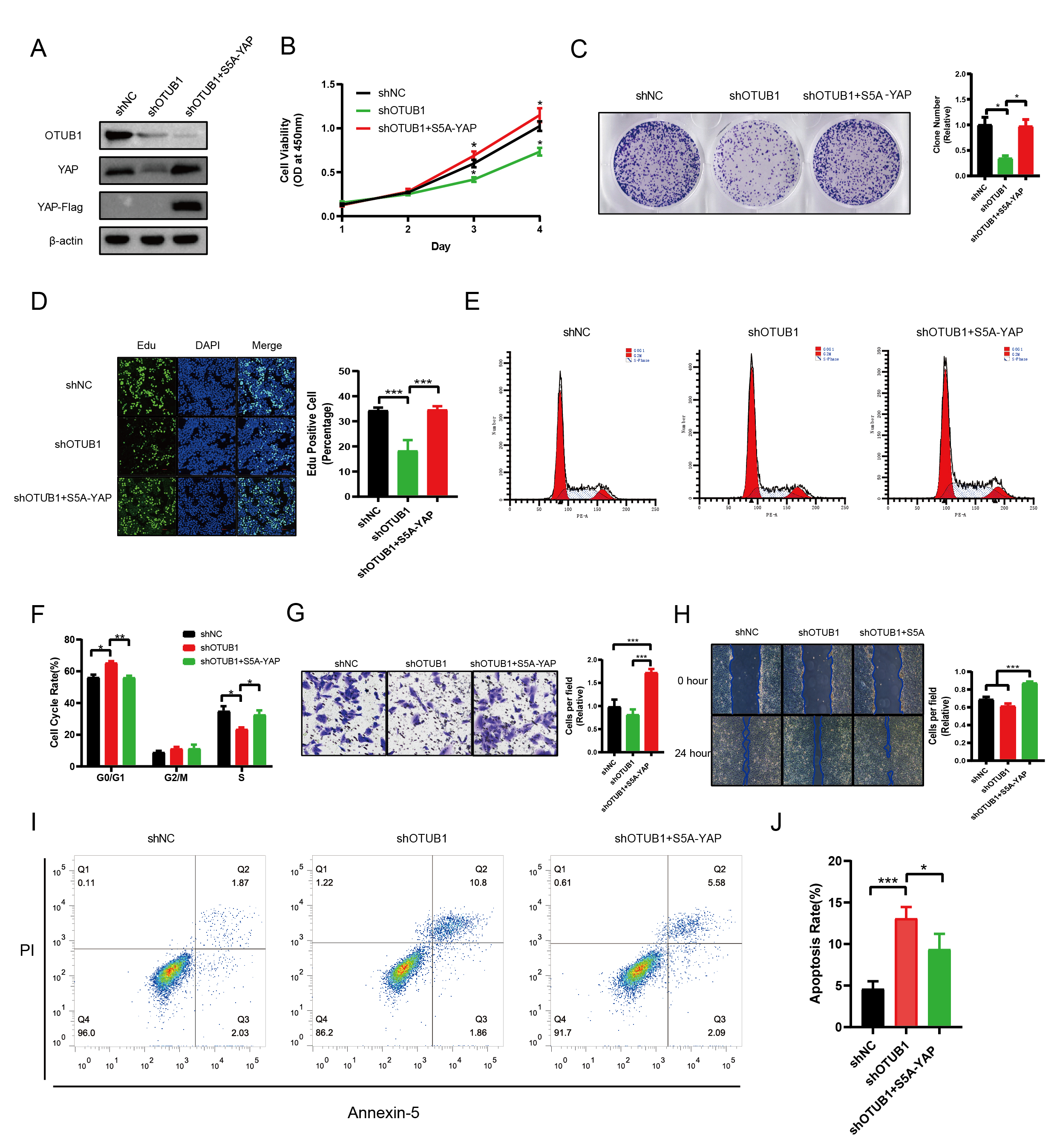

Supplement: Supplementary file 3 — Supplementary figure 3 [file 41388_2022_2507_MOESM3_ESM.jpg]

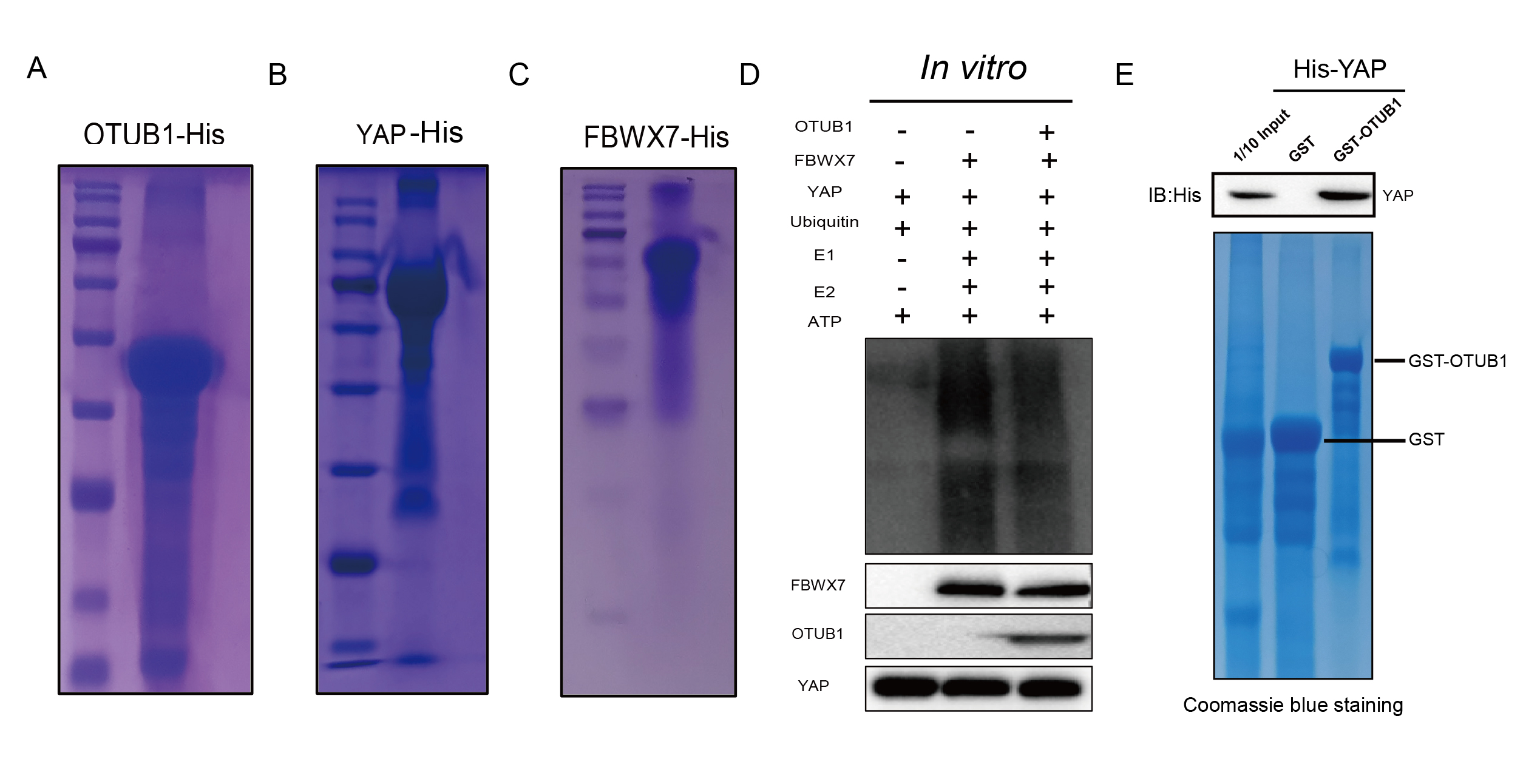

Supplement: Supplementary file 4 — Supplementary figure 4 [file 41388_2022_2507_MOESM4_ESM.jpg]
