# Supplementary material for: OTUB1 suppresses Hippo signaling via modulating YAP protein in gastric cancer
Source: Oncogene. 2022 Oct 21;41(48):5186–98. doi: 10.1038/s41388-022-02507-3 (PMC9700521; doi:10.1038/s41388-022-02507-3)
Supplement: Supplementary file 5 — Supplementary figure legends [file 41388_2022_2507_MOESM5_ESM.docx]

**Supplementary Figure 1:** Knockdown of OTUB1 doesn’t affect the expression levels of upstream components of the Hippo pathway. (A) Western blot shows protein expression of MST1, LAST1, TEAD1, TEAD2, TAZ, OTUB1, YAP, β-actin.

**Supplementary Figure 2:** Knockdown of YAP suppressed the cell proliferation, invasion and migration of gastric cancer cells induced by OTUB1 overexpression. (A-J) MKN28 cells were transduced with lentivirus expressing control or OTUB1 following with shRNAs (shScramble and shYAP). (A) Immunoblotting was performed to detect the expression of the indicated proteins. β-actin served as the loading control. (B) Cell viability was determined by CCK8 assay in the MKN28 cells transduced with lentivirus as indicated. *p < 0.05 by two-way ANOVA. (C) Colony formation (left panel) of MKN28 cells transduced with lentivirus as indicated.  Right panel shows the quantification of colony formation. *p < 0.05, **p < 0.01 by one-way ANOVA. (D) EdU assay (left panel) to show the cell proliferation of MKN28 cells transduced with lentivirus as indicated. Right panel shows the quantification of EdU assay. *p < 0.05, **p < 0.01 by one-way ANOVA. (E) Representative cell cycle plots of MKN28 cells transduced with lentivirus as indicated. (F) Quantitative summary of cell cycle analysis in MKN28 cells. *p < 0.05, **p < 0.01 by one-way ANOVA. (G) Transwell assay (left panel) of MKN28 cells transduced with lentivirus as indicated. Right panel shows quantification of transwell assay. *p < 0.05, **p < 0.01 by one-way ANOVA. (H) Wound healing assay (left panel) of KMN28 cells transduced with lentivirus as indicated. Right panel shows quantification of wound healing. **p < 0.01, ***p < 0.001 by one-way ANOVA. (I) Representative apoptosis plots of MKN28 cells. *p < 0.05, **p < 0.01 by one-way ANOVA. (J) Quantitative summary of apoptosis analysis in MKN28 cells. *p < 0.05, **p < 0.01 by one-way ANOVA.

**Supplementary Figure 3:** S5A-YAP could rescue the phenotype induced by OTUB1 depletion. (A-J) MKN28 cells were transduced with lentiviruses expressing control or shRNA targeting OTUB1, following with S5A-YAP overexpression. (A) Immunoblotting was performed to detect the expression of the indicated proteins. β-actin served as the loading control. (B) Cell growth of MKN28 cells were measured by CCK-8 assay. *p < 0.05 by two-way ANOVA. (C) Colony formation (left panel) of MKN28 cells transduced with lentivirus as indicated.  Right panel shows the quantification of colony formation. *p < 0.05, **p < 0.01 by one-way ANOVA. (D) EdU assay (left panel) to show the cell proliferation of MKN28 cells transduced with lentivirus as indicated. Right panel shows the quantification of EdU assay. *p < 0.05, **p < 0.01 by one-way ANOVA. (E) Representative cell cycle plots of MKN28 cells transduced with lentivirus as indicated. (F) Quantitative summary of cell cycle analysis in MKN28 cells. *p < 0.05, **p < 0.01 by one-way ANOVA. (G) Transwell assay (left panel) of MKN28 cells transduced with lentivirus as indicated. Right panel shows quantification of transwell assay. *p < 0.05, **p < 0.01 by one-way ANOVA. (H) Wound healing assay (left panel) of KMN28 cells transduced with lentivirus as indicated. Right panel shows quantification of wound healing. **p < 0.01, ***p < 0.001 by one-way ANOVA. (I) Representative apoptosis plots of MKN28 cells. *p < 0.05, **p < 0.01 by one-way ANOVA. (J) Quantitative summary of apoptosis analysis in MKN28 cells. *p < 0.05, **p < 0.01 by one-way ANOVA.

**Supplementary Figure 4:** OTUB1 Promotes YAP1 deubiquitination *In Vitro*. (A) Purified recombinant OTUB1. (B) Purified recombinant OTUB1. (C) Purified recombinant FBWX7. (D) In vitro ubiquitination assay was performed with E1, E2, and FBXW7 as the E3 ubiquitin ligase, together with Ub, ATP, and recombinant YAP1 as the substrate. The reaction mixture was incubated for 60 min at 30°C. (E) OTUB1 directly interacts with YAP in vitro. GST pull-down assay of purified His-YAP with the purified GST-OTUB1 from E.coli.
